# Methyl 2-(*p*-toluene­sulfonamido)benzoate

**DOI:** 10.1107/S1600536810000814

**Published:** 2010-01-13

**Authors:** Guang-You Zhang, Di-Juan Chen, Xiang-Yang Guo, Shu-Hong Wang, Jian-Guo Chang

**Affiliations:** a Department of Materials Science and Engineering, University of Jinan, Jinan 250022, People’s Republic of China; b School of Chemistry and Chemical Engineering, University of Jinan, Jinan 250022, People’s Republic of China; c Department of Materials Science and Chemical Engineering, Taishan University, Taishan 271021, People’s Republic of China

## Abstract

The title compound, C_15_H_15_NO_4_S, was prepared by simple condensation of methyl 2-amino­benzoate and 4-methyl­benzene­sulfonyl chloride. The dihedral angle between the benzene rings is 84.36 (6)°. The mol­ecular structure is stabilized by an intra­molecular N—H⋯O hydrogen-bonding inter­action involving the carbonyl group as acceptor, generating an *S*(6) graph-set motif.

## Related literature

For background information on sulfonamide derivatives and their properties, see: Sheppard *et al.* (2006 ▶). For similar structures, see: Schultz *et al.* (2001 ▶); Krishnaiah *et al.* (1995 ▶); Arshad, Khan, Shafiq *et al.* (2009 ▶); Arshad, Khan, Akkurt *et al.* (2009 ▶); Xiong *et al.* (2007 ▶).
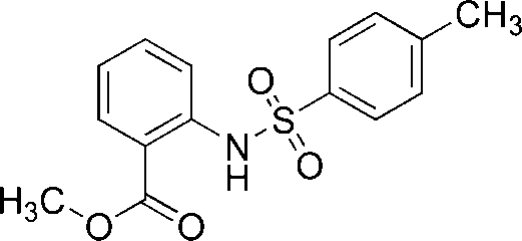


## Experimental

### 

#### Crystal data

C_15_H_15_NO_4_S
*M* *_r_* = 305.34
Monoclinic, 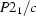

*a* = 15.0129 (13) Å
*b* = 8.3593 (7) Å
*c* = 11.9664 (11) Å
β = 96.854 (2)°
*V* = 1491.0 (2) Å^3^
*Z* = 4
Mo *K*α radiation
μ = 0.23 mm^−1^
*T* = 295 K
0.24 × 0.16 × 0.14 mm

#### Data collection

Bruker APEXII area-detector diffractometer
Absorption correction: multi-scan (*SADABS*; Sheldrick, 2003 ▶) *T*_min_ = 0.947, *T*_max_ = 0.968
7663 measured reflections
2639 independent reflections
2230 reflections with *I* > 2σ(*I*)
*R*_int_ = 0.024

#### Refinement

*R*[*F* ^2^ > 2σ(*F* ^2^)] = 0.036
*wR*(*F* ^2^) = 0.102
*S* = 1.04
2639 reflections
191 parameters
H-atom parameters constrained
Δρ_max_ = 0.15 e Å^−3^
Δρ_min_ = −0.32 e Å^−3^

### 

Data collection: *APEX2* (Bruker, 2005 ▶); cell refinement: *SAINT* (Bruker, 2005 ▶); data reduction: *SAINT*; program(s) used to solve structure: *SHELXTL* (Sheldrick, 2008 ▶); program(s) used to refine structure: *SHELXTL*; molecular graphics: *SHELXTL*; software used to prepare material for publication: *SHELXTL*.

## Supplementary Material

Crystal structure: contains datablocks I, global. DOI: 10.1107/S1600536810000814/bh2263sup1.cif

Structure factors: contains datablocks I. DOI: 10.1107/S1600536810000814/bh2263Isup2.hkl

Additional supplementary materials:  crystallographic information; 3D view; checkCIF report

## Figures and Tables

**Table 1:** Hydrogen-bond geometry (Å, °)

*D*—H⋯*A*	*D*—H	H⋯*A*	*D*⋯*A*	*D*—H⋯*A*
N1—H1⋯O2	0.87	1.89	2.640 (2)	143

**Fig. 1.**
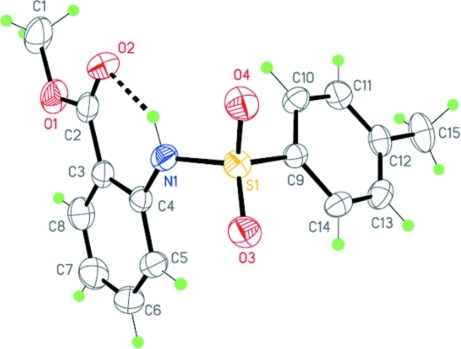
The structure of the title compound with 30% probability ellipsoids. H atoms are shown as spheres of arbitrary radii. The dashed line represents a hydrogen bond.

## supplementary crystallographic information

### Comment

Sulfur-containing compounds, such as sulfates, sulfones, thiols, sulfonamides and sulfoxides can exhibit insecticidal, germicidal or antimicrobial activities (Schultz *et al.*, 2001; Krishnaiah *et al.*, 1995; Arshad, Khan, Shafiq *et al.*, 2009; Xiong *et al.*, 2007). Particularly, the sulfonamides are biologically active organic compounds, which have been investigated as inhibitors of methionine aminopeptidase-2 (MetAP2) and intermediates for cancer therapy (Sheppard *et al.*, 2006). In order to obtain detailed information on the molecular conformation, the X-ray study of the title sulfonamide has been carried out, and the results are presented here.

As is shown in Fig. 1, the two benzene rings are approximately orthogonal, the dihedral angle between their planes being 84.36 (6)°. In addition to van der Waals interactions, there is an intramolecular N—H···O hydrogen bond which generates a graph set motif *S*(6) (Arshad, Khan, Akkurt *et al.*, 2009) to stabilize the molecular conformation (Fig. 1 and Table 1).

### Experimental

Methyl 2-aminobenzoate (5 mmol) was dissolved in tetrahydrofuran (10 ml) in a round bottom flask (50 ml). The pH of the solution was maintained at 7–8 using Et_3_N. 4-Toluene sulfonyl chloride (6 mmol) was suspended to the above solution and refluxed until all the 4-toluene sulfonyl chloride was consumed. After removal of the solvent, water (20 ml) was added to the residue. Then the mixture was extracted with CH_2_Cl_2_, and the organic layer was dried over anhydrous sodium sulfate. The solvent was removed under reduced pressure. Further purification was carried out by recrystallization in ethanol to give a solid (yield 83.7%). Single crystals suitable for X-ray analysis were obtained by diffusion of *n*-hexane in an ethanol solution.

### Refinement

All C-bonded H atoms were placed in geometrically idealized positions and constrained to ride on their parent atoms, with C—H = 0.93 (aromatic) or 0.96 Å (methyl). Amine H atom H1 was found in a difference map, and its position fixed in final cycles. Isotropic displacement parameters for H atoms were calculated as *U*_iso_(H) = 1.5*U*_eq_(carrier C, N) for methyl H atoms and H1, and *U*_iso_(H) = 1.2*U*_eq_(carrier C) for others.

### Crystal data

**Table:**

C_15_H_15_NO_4_S	*F*(000) = 640
*M**_r_* = 305.34	*D*_x_ = 1.360 Mg m^−^^3^
Monoclinic, *P*2_1_/*c*	Melting point: 380 K
Hall symbol: -P 2ybc	Mo *K*α radiation, λ = 0.71073 Å
*a* = 15.0129 (13) Å	Cell parameters from 3425 reflections
*b* = 8.3593 (7) Å	θ = 2.3–27.0°
*c* = 11.9664 (11) Å	µ = 0.23 mm^−^^1^
β = 96.854 (2)°	*T* = 295 K
*V* = 1491.0 (2) Å^3^	Block, colourless
*Z* = 4	0.24 × 0.16 × 0.14 mm

### Data collection

**Table:**

Bruker APEXII area-detector diffractometer	2639 independent reflections
Radiation source: fine-focus sealed tube	2230 reflections with *I* > 2σ(*I*)
graphite	*R*_int_ = 0.024
φ and ω scans	θ_max_ = 25.1°, θ_min_ = 2.7°
Absorption correction: multi-scan (*SADABS*; Sheldrick, 2003)	*h* = −16→17
*T*_min_ = 0.947, *T*_max_ = 0.968	*k* = −9→9
7663 measured reflections	*l* = −14→9

### Refinement

**Table:**

Refinement on *F*^2^	Primary atom site location: structure-invariant direct methods
Least-squares matrix: full	Secondary atom site location: difference Fourier map
*R*[*F*^2^ > 2σ(*F*^2^)] = 0.036	Hydrogen site location: inferred from neighbouring sites
*wR*(*F*^2^) = 0.102	H-atom parameters constrained
*S* = 1.04	*w* = 1/[σ^2^(*F*_o_^2^) + (0.0512*P*)^2^ + 0.3683*P*] where *P* = (*F*_o_^2^ + 2*F*_c_^2^)/3
2639 reflections	(Δ/σ)_max_ = 0.001
191 parameters	Δρ_max_ = 0.15 e Å^−^^3^
0 restraints	Δρ_min_ = −0.32 e Å^−^^3^

### Fractional atomic coordinates and isotropic or equivalent isotropic displacement parameters (Å^2^)

**Table:**

	*x*	*y*	*z*	*U*_iso_*/*U*_eq_	
S1	0.78611 (3)	0.16418 (5)	0.77097 (4)	0.04976 (17)	
O1	0.56582 (10)	0.0349 (2)	0.36179 (12)	0.0866 (5)	
O2	0.61287 (10)	0.2115 (2)	0.49515 (14)	0.0784 (4)	
O3	0.80078 (9)	0.05619 (17)	0.86305 (10)	0.0642 (4)	
O4	0.76453 (10)	0.32689 (16)	0.79146 (12)	0.0677 (4)	
N1	0.70193 (9)	0.10160 (18)	0.68303 (13)	0.0536 (4)	
H1	0.6798	0.1762	0.6367	0.082 (7)*	
C1	0.52814 (17)	0.1643 (4)	0.2920 (2)	0.1100 (11)	
H1A	0.4763	0.2057	0.3221	0.165*	
H1B	0.5111	0.1258	0.2169	0.165*	
H1C	0.5720	0.2477	0.2906	0.165*	
C2	0.60786 (12)	0.0751 (3)	0.46213 (17)	0.0623 (5)	
C3	0.64536 (11)	−0.0668 (2)	0.52490 (15)	0.0524 (4)	
C4	0.69256 (10)	−0.0513 (2)	0.63324 (14)	0.0474 (4)	
C5	0.72532 (13)	−0.1874 (2)	0.68995 (18)	0.0604 (5)	
H5	0.7568	−0.1781	0.7615	0.072*	
C6	0.71195 (15)	−0.3359 (2)	0.6419 (2)	0.0748 (6)	
H6	0.7343	−0.4261	0.6812	0.090*	
C7	0.66594 (16)	−0.3525 (3)	0.5364 (2)	0.0810 (7)	
H7	0.6571	−0.4534	0.5042	0.097*	
C8	0.63345 (15)	−0.2203 (3)	0.47946 (19)	0.0716 (6)	
H8	0.6023	−0.2324	0.4080	0.086*	
C9	0.88094 (11)	0.16066 (19)	0.69848 (13)	0.0436 (4)	
C10	0.88227 (13)	0.2541 (2)	0.60377 (15)	0.0556 (5)	
H10	0.8323	0.3146	0.5766	0.067*	
C11	0.95774 (14)	0.2569 (3)	0.55021 (16)	0.0626 (5)	
H11	0.9585	0.3201	0.4864	0.075*	
C12	1.03322 (12)	0.1678 (2)	0.58876 (16)	0.0581 (5)	
C13	1.03009 (13)	0.0749 (2)	0.68292 (17)	0.0623 (5)	
H13	1.0800	0.0144	0.7101	0.075*	
C14	0.95457 (12)	0.0694 (2)	0.73804 (15)	0.0541 (4)	
H14	0.9533	0.0048	0.8011	0.065*	
C15	1.11567 (15)	0.1736 (3)	0.5291 (2)	0.0877 (8)	
H15A	1.1455	0.2744	0.5438	0.132*	
H15B	1.0988	0.1620	0.4495	0.132*	
H15C	1.1555	0.0883	0.5557	0.132*	

### Atomic displacement parameters (Å^2^)

**Table:**

	*U*^11^	*U*^22^	*U*^33^	*U*^12^	*U*^13^	*U*^23^
S1	0.0530 (3)	0.0496 (3)	0.0468 (3)	−0.00029 (19)	0.00665 (19)	−0.00452 (18)
O1	0.0675 (9)	0.1255 (14)	0.0618 (9)	−0.0107 (9)	−0.0128 (7)	0.0207 (9)
O2	0.0743 (10)	0.0717 (10)	0.0861 (11)	0.0118 (8)	−0.0036 (8)	0.0221 (9)
O3	0.0755 (9)	0.0734 (9)	0.0435 (7)	−0.0095 (7)	0.0067 (6)	0.0063 (6)
O4	0.0695 (9)	0.0557 (8)	0.0801 (9)	0.0029 (6)	0.0177 (7)	−0.0180 (7)
N1	0.0498 (8)	0.0470 (8)	0.0620 (9)	0.0048 (7)	−0.0016 (7)	−0.0006 (7)
C1	0.0695 (15)	0.170 (3)	0.0853 (17)	−0.0053 (16)	−0.0123 (13)	0.0621 (19)
C2	0.0391 (9)	0.0881 (16)	0.0591 (12)	−0.0040 (10)	0.0037 (8)	0.0139 (11)
C3	0.0395 (9)	0.0634 (11)	0.0542 (10)	−0.0029 (8)	0.0053 (7)	0.0010 (9)
C4	0.0382 (8)	0.0497 (10)	0.0549 (10)	0.0001 (7)	0.0078 (7)	−0.0011 (8)
C5	0.0592 (11)	0.0540 (11)	0.0657 (12)	0.0030 (9)	−0.0018 (9)	0.0046 (9)
C6	0.0740 (14)	0.0486 (12)	0.1004 (17)	0.0041 (10)	0.0046 (13)	0.0025 (11)
C7	0.0807 (15)	0.0570 (13)	0.1052 (19)	−0.0042 (11)	0.0099 (14)	−0.0210 (13)
C8	0.0667 (13)	0.0823 (15)	0.0642 (13)	−0.0117 (11)	0.0007 (10)	−0.0163 (11)
C9	0.0478 (9)	0.0432 (9)	0.0384 (8)	0.0004 (7)	−0.0004 (7)	−0.0031 (7)
C10	0.0557 (11)	0.0626 (12)	0.0470 (10)	0.0063 (9)	0.0004 (8)	0.0076 (8)
C11	0.0692 (13)	0.0724 (14)	0.0463 (10)	−0.0107 (10)	0.0077 (9)	0.0042 (9)
C12	0.0525 (11)	0.0652 (12)	0.0572 (11)	−0.0128 (9)	0.0091 (9)	−0.0235 (9)
C13	0.0495 (11)	0.0650 (12)	0.0705 (13)	0.0103 (9)	−0.0010 (9)	−0.0100 (10)
C14	0.0588 (11)	0.0532 (11)	0.0484 (10)	0.0087 (8)	−0.0013 (8)	0.0033 (8)
C15	0.0654 (14)	0.109 (2)	0.0933 (17)	−0.0258 (13)	0.0296 (12)	−0.0401 (15)

### Geometric parameters (Å, °)

**Table:**

S1—O3	1.4212 (13)	C6—H6	0.9300
S1—O4	1.4262 (14)	C7—C8	1.358 (3)
S1—N1	1.6311 (15)	C7—H7	0.9300
S1—C9	1.7535 (17)	C8—H8	0.9300
O1—C2	1.331 (2)	C9—C10	1.379 (2)
O1—C1	1.440 (3)	C9—C14	1.380 (2)
O2—C2	1.206 (3)	C10—C11	1.367 (3)
N1—C4	1.410 (2)	C10—H10	0.9300
N1—H1	0.8734	C11—C12	1.388 (3)
C1—H1A	0.9600	C11—H11	0.9300
C1—H1B	0.9600	C12—C13	1.374 (3)
C1—H1C	0.9600	C12—C15	1.502 (3)
C2—C3	1.479 (3)	C13—C14	1.379 (3)
C3—C8	1.397 (3)	C13—H13	0.9300
C3—C4	1.407 (2)	C14—H14	0.9300
C4—C5	1.385 (2)	C15—H15A	0.9600
C5—C6	1.373 (3)	C15—H15B	0.9600
C5—H5	0.9300	C15—H15C	0.9600
C6—C7	1.372 (3)		
			
O3—S1—O4	119.33 (9)	C8—C7—C6	119.4 (2)
O3—S1—N1	109.45 (8)	C8—C7—H7	120.3
O4—S1—N1	104.03 (8)	C6—C7—H7	120.3
O3—S1—C9	108.24 (8)	C7—C8—C3	122.0 (2)
O4—S1—C9	108.40 (8)	C7—C8—H8	119.0
N1—S1—C9	106.73 (8)	C3—C8—H8	119.0
C2—O1—C1	116.4 (2)	C10—C9—C14	120.39 (17)
C4—N1—S1	126.43 (12)	C10—C9—S1	119.30 (13)
C4—N1—H1	111.6	C14—C9—S1	120.27 (13)
S1—N1—H1	112.8	C11—C10—C9	119.29 (17)
O1—C1—H1A	109.5	C11—C10—H10	120.4
O1—C1—H1B	109.5	C9—C10—H10	120.4
H1A—C1—H1B	109.5	C10—C11—C12	121.63 (18)
O1—C1—H1C	109.5	C10—C11—H11	119.2
H1A—C1—H1C	109.5	C12—C11—H11	119.2
H1B—C1—H1C	109.5	C13—C12—C11	117.99 (17)
O2—C2—O1	122.6 (2)	C13—C12—C15	121.3 (2)
O2—C2—C3	125.93 (19)	C11—C12—C15	120.7 (2)
O1—C2—C3	111.5 (2)	C12—C13—C14	121.46 (18)
C8—C3—C4	118.07 (18)	C12—C13—H13	119.3
C8—C3—C2	121.06 (18)	C14—C13—H13	119.3
C4—C3—C2	120.86 (17)	C13—C14—C9	119.23 (17)
C5—C4—C3	119.06 (17)	C13—C14—H14	120.4
C5—C4—N1	121.75 (16)	C9—C14—H14	120.4
C3—C4—N1	119.14 (16)	C12—C15—H15A	109.5
C6—C5—C4	120.82 (19)	C12—C15—H15B	109.5
C6—C5—H5	119.6	H15A—C15—H15B	109.5
C4—C5—H5	119.6	C12—C15—H15C	109.5
C7—C6—C5	120.6 (2)	H15A—C15—H15C	109.5
C7—C6—H6	119.7	H15B—C15—H15C	109.5
C5—C6—H6	119.7		
			
O3—S1—N1—C4	−53.87 (17)	C6—C7—C8—C3	0.0 (4)
O4—S1—N1—C4	177.55 (15)	C4—C3—C8—C7	−0.1 (3)
C9—S1—N1—C4	63.05 (16)	C2—C3—C8—C7	−178.8 (2)
C1—O1—C2—O2	1.7 (3)	O3—S1—C9—C10	178.84 (13)
C1—O1—C2—C3	−178.72 (17)	O4—S1—C9—C10	−50.38 (16)
O2—C2—C3—C8	178.3 (2)	N1—S1—C9—C10	61.13 (15)
O1—C2—C3—C8	−1.3 (2)	O3—S1—C9—C14	−3.30 (16)
O2—C2—C3—C4	−0.4 (3)	O4—S1—C9—C14	127.47 (15)
O1—C2—C3—C4	−179.97 (15)	N1—S1—C9—C14	−121.02 (14)
C8—C3—C4—C5	0.2 (3)	C14—C9—C10—C11	−0.8 (3)
C2—C3—C4—C5	178.93 (16)	S1—C9—C10—C11	177.06 (14)
C8—C3—C4—N1	−177.13 (16)	C9—C10—C11—C12	0.1 (3)
C2—C3—C4—N1	1.6 (2)	C10—C11—C12—C13	0.3 (3)
S1—N1—C4—C5	34.0 (2)	C10—C11—C12—C15	−179.53 (18)
S1—N1—C4—C3	−148.77 (14)	C11—C12—C13—C14	0.1 (3)
C3—C4—C5—C6	−0.2 (3)	C15—C12—C13—C14	179.90 (18)
N1—C4—C5—C6	177.03 (18)	C12—C13—C14—C9	−0.8 (3)
C4—C5—C6—C7	0.1 (3)	C10—C9—C14—C13	1.2 (3)
C5—C6—C7—C8	0.0 (4)	S1—C9—C14—C13	−176.68 (14)

### Hydrogen-bond geometry (Å, °)

**Table:**

*D*—H···*A*	*D*—H	H···*A*	*D*···*A*	*D*—H···*A*
N1—H1···O2	0.87	1.89	2.640 (2)	143
